# Identification of multi-drug resistant *Acinetobacter baumannii* phage YZ2 and evaluation of its therapeutic efficacy *in vivo* and *in vitro*

**DOI:** 10.3389/fmicb.2025.1657539

**Published:** 2025-11-24

**Authors:** Yaru Zhi, Dongmei Yan, Qinfang Tang, Lihua Xiao, Aiting Cai, Mingzhong Sun, Hongmei Chen, Yungang Wang, Qingping Fu

**Affiliations:** Department of Laboratory Medicine, Affiliated Hospital 6 of Nantong University, Yancheng Third People's Hospital, Yancheng, China

**Keywords:** *Acinetobacter baumannii*, multidrug resistant, phage YZ2, genome analysis, biological characteristics, phage therapy

## Abstract

**Introduction:**

Carbapenem-resistant *Acinetobacter baumannii* (CRAB) has recently become an important pathogen in clinically acquired infections, making treatment more challenging.

**Methods:**

The treatment of bacterial infections may improve with the development of phage therapy and phage-antibiotic combination therapy. Here, we reported a novel phage YZ2 that has a double-stranded DNA genome of 40,181 bp with 37.93% GC content. A total of 46 open reading frames (ORFs) and no virulence or antimicrobial resistance genes were annotated in the genome of phage YZ2. Phage YZ2 is a novel member of the *Autographiviridae*, with a latency period of approximately 20 min and a burst size of approximately 134 phage particles per infected host cell.

**Results:**

The *in vitro* antibacterial results demonstrated that YZ2 could rapidly eliminate host bacteria at a low multiplicity of infection, showing strong bactericidal efficacy. *In vivo*, YZ2 significantly increased the survival rate of *A. baumannii*-infected *Galleria mellonella* larvae from 10 to 100% within 72 h.

**Discussion:**

Moreover, compared with the use of phage or polymyxin B alone, the combined use of phage YZ2 and polymyxin B can significantly increased the survival rate of *G. mellonella* larvae and had a synergistic effect. These results imply that phage YZ2 has the potential for development as an antimicrobial agent.

## 1 Introduction

*Acinetobacter baumannii* (*A. baumannii*), a notorious ESKAPE pathogen (Kyriakidis et al., 2021), has emerged as a critical global public health threat, designated by the World Health Organization as Priority 1: a critical pathogen posing the highest risk to human health (Sati et al., 2025). The severity of this threat is underscored by the fact that in 2021, approximately 90% of deaths related to *A. baumannii* infections worldwide were caused by drug-resistant strains. In many regions, such as India, Southeast Asia, the Middle East, and South America, the rate of carbapenem-resistant *Acinetobacter baumannii* (CRAB) has exceeded 50% (Dubey et al., 2025). Global monitoring data reveals that its prevalence remains high and continues to evolve, with significant annual increases observed in both Europe and the United States (Wise et al., 2024). In China, the situation is particularly severe, with the CRAB isolation rate reaching approximately 70% and continuing to rise (Luo et al., 2024a). This high baseline resistance, combined with its tendency towards pan-resistance (typically only sensitive to polymyxins and tigecycline), makes carbapenem drugs often ineffective at the start of treatment, posing a significant clinical challenge. In addition to the high baseline drug resistance, what is even more alarming is its rapid adaptive evolution during the treatment process. Studies have shown that *A. baumannii* can evolve within the host and rapidly increase its drug resistance level under the pressure of antibacterial drugs, resulting in the loss of drug sensitivity during the treatment and being one of the key factors causing clinical treatment failure (Hua et al., 2017). *A. baumannii* shows continuously increasing resistance to carbapenems worldwide, which has become a serious challenge in ICU infections (Nordmann and Poirel, 2019). Studies demonstrate that clinical isolates of *A. baumannii* have developed resistance to nearly all potent antimicrobial agents in use (Larsson and Flach, 2021), leading to the emergence of extensively drug-resistant and pan drug-resistant strains. This severely limits the therapeutic options and significantly increases patient morbidity and mortality rates.

International clone (IC) 2 is the most prevalent CRAB strain worldwide (Hamidian and Nigro, 2019; Wang et al., 2024a). Oxford sequence types (STs) 195, 191, and 208 are most prevalent lineages of *A. baumannii* IC2. Among them, ST208 is a widely prevalent and concerning lineage, with its proportion significantly increasing (from 12.93% in 2014 to 21.19% in 2021). Its high virulence and high drug resistance pose serious threats to clinical treatment (Luo et al., 2025). Meanwhile, emerging lineages such as ST540 are becoming increasingly prominent in specific geographical regions, such as China, highlighting the dynamic and evolving nature of CRAB populations (Huang et al., 2024; Luo et al., 2025).

Colistin remains the only effective antibiotic for treating carbapenem-resistant *A. baumannii* (CRAB) infections (Cruz-López et al., 2022). However, colistin therapy carries the risks of nephrotoxicity and neurotoxicity, with higher doses correlating with greater toxicity (Gunay et al., 2020). The inappropriate application of colistin in both clinical and veterinary settings has accelerated the emergence of drug-resistant bacterial variants at an accelerated rate (El-Sayed Ahmed et al., 2020). Consequently, it is imperative to reduce the use of antibiotics and explore alternative treatments.

Bacteriophages (phages) are specialized viruses that infect and disrupt bacterial cells(Zhu et al., 2024). These viruses outnumber all other biological entities in the natural environment (Tu et al., 2023) and are regarded as promising therapeutic alternatives in the post-antibiotic era (Elshamy et al., 2025). To date, multiple multidrug-resistant (MDR) *A. baumannii*-specific phages have been successfully isolated and characterized (Oliveira et al., 2022; Alseth et al., 2025). For example, the phage vABPW7 demonstrated potent antibacterial efficacy against MDR *A. baumannii* in a human A549 alveolar epithelial cell mode (Wintachai et al., 2022b). Phage therapy is highly specific, efficient, and stable. However, the high host specificity of phages and the emergence of phage-resistant bacteria also limit the application of single phage therapy (Tu et al., 2023; Uchechukwu and Shonekan, 2024). To address this, the application of phage cocktails has been shown to significantly reduce the evolution of drug-resistant bacteria (Dąbrowska and Abedon, 2019; Wang et al., 2024c). Moreover, phage-antibiotic combinations can synergistically enhance therapeutic outcomes (Domingo-Calap and Delgado-Martínez, 2018). Studies have reported that different phage-antibiotic pairings may exhibit synergistic or antagonistic interactions *in vitro* (Gu Liu et al., 2020; Wang et al., 2024b). The synergistic effects of phage cocktails and antibiotics have been validated in a human urine model (Grygorcewicz et al., 2021). Preclinical evidence further supports the idea that phage-antibiotic combination therapy represents an optimal strategy against *A. baumannii* infection (Gordillo Altamirano et al., 2022). Therefore, to prepare safe and effective phage cocktails or to implement synergistic phage-antibiotic combinations, it is particularly important to isolate new bacteriophages and conduct detailed analyses on them (Schooley et al., 2017). Although several phages targeting *A. baumannii* have been reported, there is still an urgent clinical need for new phages that can exert a synergistic effect with existing ‘last line of defense’ antibiotics (such as polymyxin B) to enhance efficacy and reduce antibiotic toxicity.

This study reports the isolation and characterization of phage YZ2, a novel CRAB virus. The complete genome was sequenced, and its critical biological properties were systematically investigated. In addition, we evaluated the therapeutic effect of phage combined with polymyxin B in the *Galleria mellonella* (*G. mellonella*) larvae model, which provides a useful reference for future clinical treatment.

## 2 Materials and methods

### 2.1 Source of strains and growth conditions

In this study, 26 strains of clinically CRAB were isolated from patient specimens at Yancheng Third People’s Hospital. Additionally, reference strains, including *Escherichia coli* ATCC 25922, *Klebsiella pneumoniae* ATCC 700603, and *Pseudomonas aeruginosa* ATCC 27853 (maintained as laboratory stock cultures), were selected to determine the host range of the bacteriophages.

To elucidate the molecular epidemiological characteristics of CRAB isolates, multilocus sequence typing was performed on the strains according to the Oxford protocol (Jolley et al., 2018). Notably, the CRAB strain AB0814 (the host strain for bacteriophage YZ2, GenBank accession number: PRJNA1276704), which was isolated in 2022 from the urine specimen of a patient with a hospital-acquired urinary tract infection in the ICU of Yancheng Third People’s Hospital, was specifically selected for bacteriophage YZ2 preparation, characterization of biological properties, and evaluation of therapeutic efficacy *in vivo*. Multilocus sequence typing revealed that AB0814 belongs to ST540, a carbapenem-resistant sequence type that is predominantly prevalent in China and highly clinically relevant (Luo et al., 2025). Antimicrobial susceptibility testing against a panel of antibiotics was performed using the broth microdilution method according to CLSI guidelines (CLSI, 2023). This strain exhibits characteristics of multidrug resistance, including resistance to various antibiotics such as imipenem and meropenem, and its antibacterial spectrum is shown in Supplementary Table 1.

All clinical isolates were identified and confirmed using the VITEK 2 Compact Automated Microbial Identification System. Duplicate strains were excluded through reproducibility testing. Bacterial cultivation was conducted using Luria-Bertani (LB) medium: the liquid medium contained 10 g/L NaCl, 10 g/L tryptone, and 5 g/L yeast extract, whereas the solid medium was supplemented with 1.5% (w/v) agar. All cultures were maintained at 37 °C (±0.5 °C) with continuous temperature monitoring to ensure optimal growth conditions. For long-term preservation, bacterial stocks were prepared in LB liquid medium containing 20% glycerol and stored at −70 °C.

### 2.2 Isolation and purification of bacteriophages

Bacteriophage isolation was performed using the CRAB clinical strain AB0814 as the host bacterium. Untreated wastewater (20 mL) was collected from the Sewage Treatment Center of Yancheng Third People’s Hospital. Following centrifugation at 5,000 rpm for 10 min, the sample was filtered through a 0.22 μm membrane (Biosharp, China) to collect the supernatant. Subsequently, 20 mL of LB broth and 400 μL of log-phase AB0814 culture were added to the supernatant. After overnight incubation at 37 °C with 200 rpm agitation, the enriched culture was centrifuged (5,000 rpm, 10 min, 4 °C) and sterile-filtered (0.22 μm pore size) to obtain aseptic supernatant. A 100 μL aliquot of the supernatant was mixed with 100 μL of stationary-phase AB0814 culture, combined with 3.5 mL of soft agar (0.6% agar), and overlaid onto solid agar plates. The plates were incubated at 37 °C overnight. Phage plaques were observed the next day, and individual plaques were excised for further purification. The dual-agar overlay technique was performed through at least three successive infection cycles to isolate phages forming plaques of uniform diameter.

### 2.3 Host range analysis of phage YZ2

The host specificity of YZ2 against different bacterial strains was examined using a standard spot assay (Kutter, 2009). Briefly, 100 μL logarithmic-phase bacterial culture was combined with 3.5 mL melted soft agar (0.6%) and poured onto pre-prepared LB agar plates. Following solidification, each test strain’s lawn was spotted with 5 μL aliquots of serially diluted YZ2 suspensions (10^9^–10^3^ plaque-forming units per milliliter [PFU/mL]). To ascertain strain susceptibility to YZ2, bacterial growth inhibition was assessed after an overnight incubation at 37 °C.

### 2.4 Efficiency of plating (EOP)

EOP method has been previously detailed by Kutter (Li et al., 2023). The phage was plated on double-layer agar plates with clinical strains that tested positive in standard spot tests. Relative EOP was calculated as the ratio of the number of plaques on each clinical strain to the number of plaques on the host strain after counting the number of plaques on each plate.

### 2.5 Morphological observation of phage YZ2

The morphology of purified phage YZ2 (10^11^PFU/mL) was observed by transmission electron microscopy (TEM). Briefly, a suspension of phage YZ2 was coated onto a carbon-coated copper mesh and allowed to adsorb for 2 min. Negative staining was performed with 2% (w/v) phosphotungstic acid for 2 min and then immediately dried with filter paper. After drying in air at 25 °C, the morphology of the phages was examined using an 80 kV TEM (JEM-1400plus, JEOL, Japan).

### 2.6 Optimal multiplicity of infection

Using SM buffer, a series of 10-fold serial dilutions of the phage were prepared to determine the best multiplicity of infection (MOI). Upon growth to the log phase, phages and host bacterium *A. baumannii* AB 0814 were combined at MOI ratios of 0.001, 0.01, 0.1, 1, and 10. After 5 min of incubation at 37 °C, the mixtures were spun up for a minute at 12,000 rpm in order to eliminate any unadsorbed phages by discarding the supernatant. After precipitating and resuspending the bacteria in 5 mL of LB liquid medium, they were shaken for 2 h at 200 rpm at 37 °C. To measure the phage titer, the culture was centrifuged at 12,000 rpm, the supernatant was filtered, and a double-layer agar plaque assay was performed. PFU/mL were used to calculate the results.

### 2.7 One-step growth curve

*A. baumannii* AB0814 was cultured to the logarithmic growth phase using the one-step growth curve method and infected with phage YZ2 at an MOI of 0.1. The infected culture was incubated at 37 °C, shaken at 200 rpm, and samples were collected every 5 min for a maximum of 70 min. The bacteriophage titer in the supernatant was determined using the double-layer AGAR blank spot method. The growth curve was helpful in determining the incubation period, outbreak period, and outbreak size of bacteriophages (Pajunen et al., 2000; Kropinski, 2018).

### 2.8 Determination of thermal and pH stability of bacteriophages

For the thermal stability testing of bacteriophage YZ2, the phage was incubated at various temperatures (4, 37, 40, 50, 60, 70, and 80 °C) for 1 and 2 h, respectively, followed by gradual cooling on ice. Phage stability and viability were assessed by the double-layer agar plate method.

For pH stability testing, the LB medium was adjusted to pH 2–12 using HCl or NaOH. A 500 μL aliquot of YZ2 phage suspension (5 × 10^9^ PFU/mL) was added to LB liquid medium at each pH level and incubated at 37 °C for 1 h. Phage titers were determined using the double-layer agar plate method (Kropinski et al., 2009).

### 2.9 Determination of chloroform sensitivity

Chloroform sensitivity testing serves as a diagnostic tool for phage lipid content, as virion-associated lipids in the capsid or tail structures typically render phages susceptible to organic solvent inactivation. Phage stability was evaluated after 30 min exposure to 0–5% chloroform at physiological temperature, and viability was assessed through plaque formation on host lawns.

### 2.10 Genome extraction, sequencing, and analysis of phage YZ2

Phage preparations were sequentially treated with DNase I and RNase A (37 °C, 2 h) to remove exogenous nucleic acids, followed by proteinase K digestion (55 °C, 15 min; Tiangen Biotech). Viral DNA was purified using a TIANamp Virus DNA Kit (Tiangen Biotech).

Genome sequencing was performed using the Illumina NovaSeq PE150 platform. Raw sequencing data were processed using the following bioinformatics pipeline: (1) contaminant sequences were removed using BBMap v38.51; (2) phage genome assembly was performed using SPAdes (v3.14.1) and SOAPdenovo (v2.04) for comparative analysis; and (3) the resulting contigs were subjected to BLAST analysis (v2.10.0+) against the virus-NT database for sequence homology identification.

Functional gene annotation was performed using Prokka (v1.14.5) (Seemann, 2014), followed by protein sequence annotation using NCBI BLAST tools. Protein physicochemical properties were predicted using the ExPASy ProtParam tool by calculating the theoretical molecular weights and isoelectric points of all putative Open reading frame (ORF) products (Karunarathna et al., 2020). Genomic screening for virulence factors and antibiotic resistance genes was performed using the CGE tools VirulenceFinder^2^ and ResFinder (https://cge.food.dtu.dk/services/ResFinder) with default parameters and minimum identity thresholds of 90% (Bortolaia et al., 2020). The complete genome of phage YZ2 was constructed using the CGView Server^3^ and comparative genomic analysis at the DNA level with closely related phages was conducted using EasyFig.

Phylogenetic trees were constructed based on the major capsid protein (MCP), DNA polymerase I, endolysin, and holin proteins of the bacteriophages. The four genes were analyzed by comparison with other phage sequences in the NCBI database using BLASTX. Subsequently, a phylogenetic tree was constructed with 1,000 bootstrap replicates using the neighbor-joining (NJ) method in the Molecular Evolutionary Genetics Analysis software (MEGA), version 7.0.

### 2.11 Phage nucleotide sequence accession number

Phage YZ2’s genomic sequence is available in GenBank with the accession number OR660046.

### 2.12 Assessment of the bacteriostatic effect of phages

An overnight culture of *A. baumannii* AB0814 was transferred to 5 mL of LB liquid medium in a shaking flask at a 1:100 ratio. The culture was incubated at 37 °C with shaking at 200 rpm until the OD_600_ was approximately 0.3. Then, phages and host bacteria were combined at MOI ratios of 0.01, 0.1, 1, and 10, respectively, an uninfected bacterial cultures were used as controls. These mixtures were incubated at 37 °C with shaking at 200 rpm. Further, 200 μL samples were collected every hour and bacterial OD_600_ was measured using an Eppendorf BioSpectrometer® basic (Eppendorf China). The cultures were monitored continuously for 8 h and each experimental group was repeated in triplicate.

### 2.13 Assessment of therapeutic efficacy in *G. Mellonella* larvae

To eliminate endotoxins from *A. baumannii*, phage suspensions were prepared according to previously described methods with minor modifications (Hietala et al., 2019). Briefly, 1 M NaCl was added to 50 mL of the YZ2 lysate and incubated on ice for 1 h. A filter with a pore size of 0.22 μm was used. PEG 8000 was added with a final concentration of 10% (m/v) to the supernatant, stirred until completely dissolved, and incubated overnight at 4 °C. The supernatant was collected and centrifuged at 10,000 rpm for 10 min at 4 °C. After discarding the supernatant, the pellet was resuspended in 1 mL SM Buffer. After extracting the solution twice with 1 mL of chloroform, it was centrifuged at 10,000 rpm for 20 min at 4 °C. The final step involved filtering the mixture through a 0.22 μm pore-size filter to obtain the phage suspension. The endotoxin level in the purified phage solution was quantified using the kinetic chromogenic limulus amebocyte lysate (LAL) assay at Nanjing Kingmed Clinical Laboratory.

The injections were performed as previously described(Li et al., 2023). *Galleria mellonella* larvae, obtained from Baiyun Industrial Co., Ltd. (Jiyuan, Henan Province, China), were used as a model to test the anti-infective efficacy of the phages. Larvae with an average weight of approximately 300 mg and active movement were selected. To determine the appropriate infection dose, a microsyringe was used to inject 10 μL of *A. baumannii* AB0814 host bacteria at different concentrations into the last left proleg.

To evaluate the therapeutic efficacy of the phages, *A. baumannii* AB0814 was freshly cultured, washed with phosphate-buffered saline (PBS), and diluted to a concentration of 5 × 10^7^ CFU/mL. Ten μL of *A. baumannii* AB0814 suspension was injected into the distal end of the left forelimb of the *G. mellonella* larva of the large wax moth. Twenty minutes after infection, 10 μL of purified phage suspension was injected on the other side of the infected site, with MOI of 0.1, 1 and 10, respectively. All *G. mellonella* larvae were incubated at 37 °C throughout the experiment to better mimic the mammalian host’s physiological temperature and assess therapeutic efficacy under clinically relevant conditions.

AB0814 is resistant to multiple antibiotics but susceptible to polymyxins. Polymyxin B (RUIBIO, China) at a concentration of 2.5 mg/kg was used to treat larvae injected with the same concentration of bacterial cells (Hornsey and Wareham, 2011). Similarly, phages and polymyxin B were combined to treat *G. mellonella* larvae infected with AB0814. Following clinical dosage guidelines, each larva was injected with 10 μL of the antibiotic, resulting in a final polymyxin B concentration of 2.5 mg/kg. The larvae of the wax borer in the positive control group were infected with *A. baumannii* AB0814. The negative controls included larvae injected with phages at an MOI of 10 and larvae injected with PBS alone. Each group had 10 larvae, and the experiment was repeated in triplicate. Larval viability was assessed every 12 h over a 72-h observation period. Larvae that did not respond to contact and whose bodies turned black were marked dead.

### 2.14 Statistical analysis

All statistical analyses were performed using GraphPad Prism (version 9.1.0). Between-group comparisons were performed using the *t-test*, with *p*-values <0.05 considered statistically significant.

## 3 Results

### 3.1 Isolation and morphologic observation of phage

**Figure 1:**
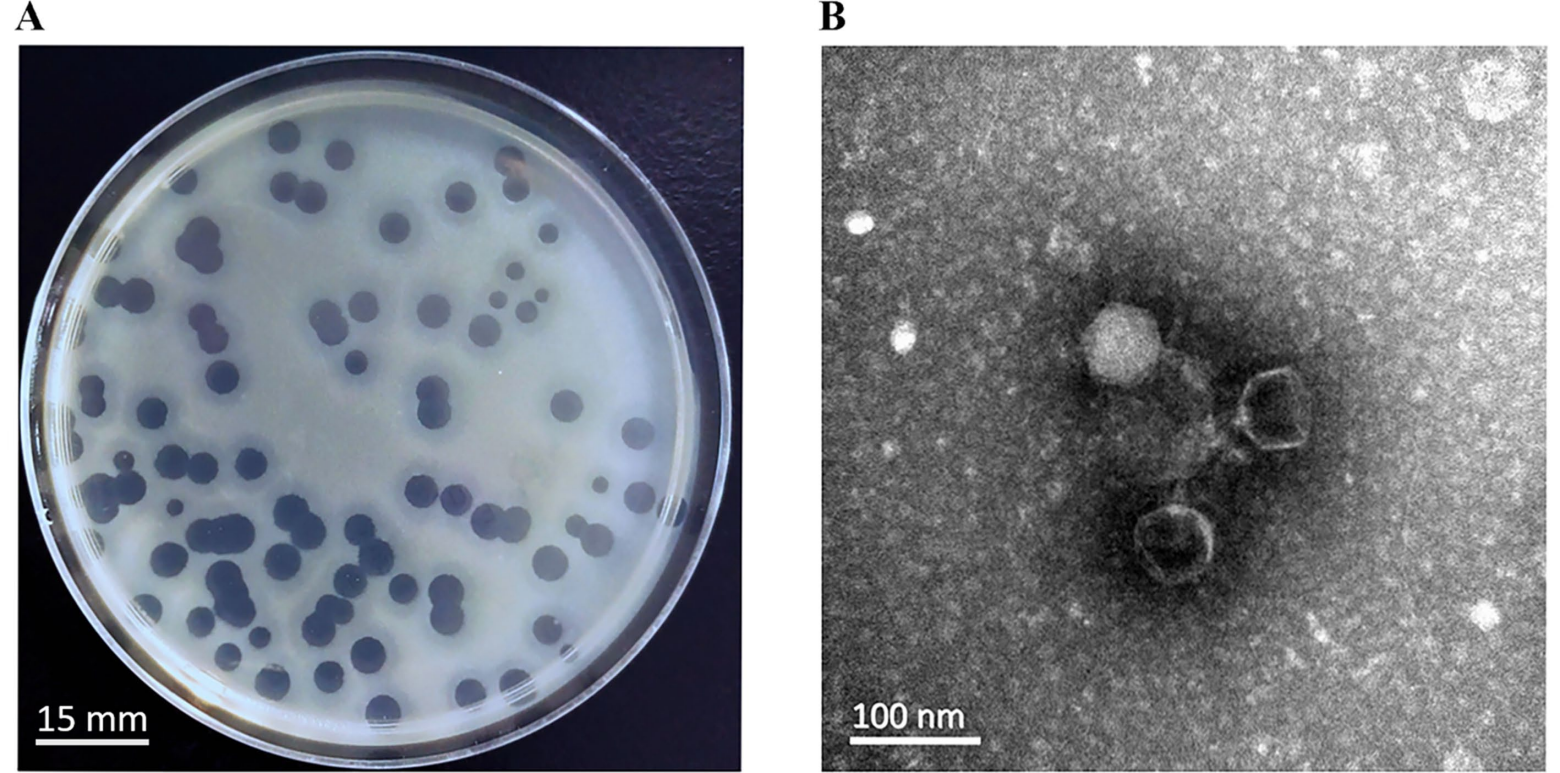
Morphological characteristics of phage YZ2. **(A)** Plaques of phage YZ2 were formed on the lawn of host strain AB0814 following 18 h of incubation at 37 °C. The plaque assay was performed using LB plates containing 1.5% agar as the bottom layer and 0.6% agar as the top layer. The scale bar represents 15 mm. **(B)** Transmission electron microscopy (TEM) image of phage YZ2. The micrograph reveals that YZ2 possesses an icosahedral head and a short tail. The scale bar represents 100 nm.

A novel lytic phage, designated YZ2, was isolated from sewage samples at the Third People’s Hospital of Yancheng City using ST540-type CRAB AB0814 as the host strain. In the double-layer agar plaque assay, after three rounds of purification, the phage formed uniform, round, clear plaques 5.0 mm in diameter, and plaque-surrounding halos were observed on double-layer plates (Figure 1A). Electron micrographs demonstrated YZ2’s characteristic morphology—an icosahedral capsid (62 ± 3 nm diameter) coupled with a short non-contractile tail (22 ± 2 nm) (Figure 1B, scale bar: 100 nm), and characteristic icosahedral head morphology and tail architecture. Taxonomic identification using PhaBOX confirmed this morphological classification and clearly assigned its genome to the *Friunavirus* genus within the *Autographiviridae* family (Shang et al., 2023). Stability of Phage YZ2.

**Figure 2:**
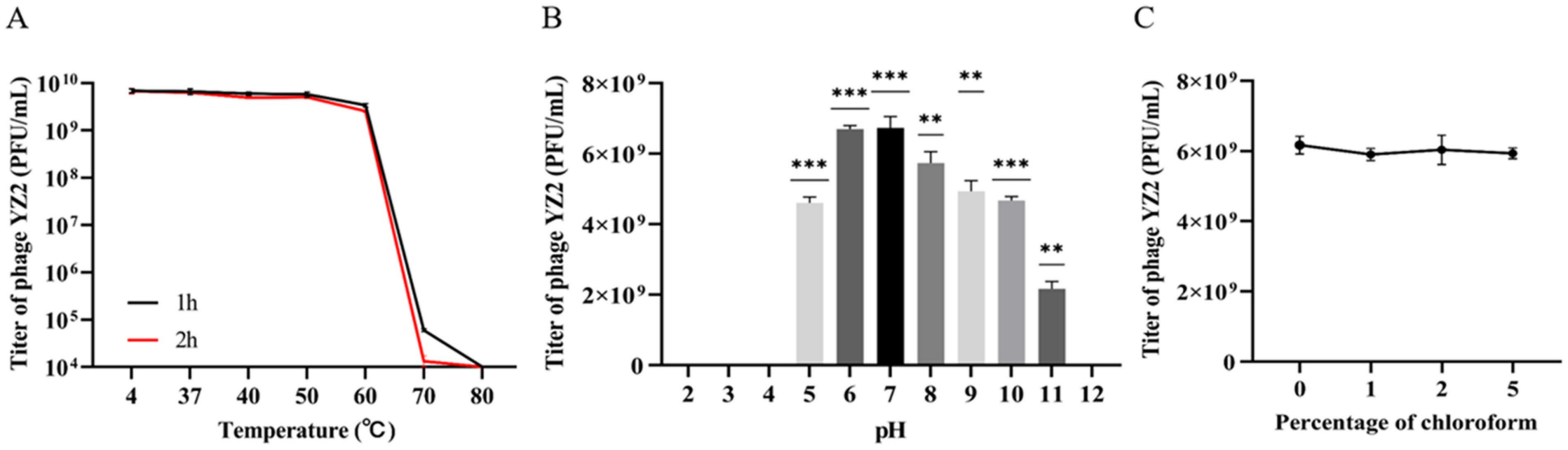
Stability of Phage YZ2. **(A)** Phage YZ2’s stability at various temperatures; **(B)** Phage YZ2’s stability across different pH levels; **(C)** Phage YZ2’s reaction to chloroform. Each experiment was conducted three times independently, and the data are shown as mean ± SD (*p < 0.05, **p < 0.01, ***p < 0.001).

The stability of phage YZ2 was evaluated under various temperature and pH conditions to confirm its clinical relevance. The phage’s ability to withstand temperatures of 4 °C, 37 °C, 40 °C, 50 °C, 60 °C, 70 °C, and 80 °C was evaluated. Findings showed that the phage titer began to decrease at 60 °C, with significantly reduced viability at higher temperatures (>60 °C). However, YZ2 demonstrated excellent stability between 4 °C and 60 °C, suggesting favorable storage conditions (Figure 2A).

For pH stability testing, the phage maintained stable lytic activity within the pH range of 5.0–11.0. YZ2 activity diminished with further pH fluctuations and decreased sharply at pH 11. No activity was detected at pH 2, 3, 4, or 12 (Figure 2B), indicating that extreme pH levels may compromise phage stability.

The protein capsid of a bacteriophage may contain lipids that play key role in inserting the viral genome into host cells (Peralta et al., 2013). To investigate whether bacteriophage YZ2 contains lipids, phage YZ2 was exposed to increasing chloroform concentrations (0–5% w/v) at 37 °C for 30 min, followed by plaque assay to determine residual infectivity. The results showed that, following exposure to varying levels of chloroform, the viability of phage YZ2 did not change significantly (Figure 2C). Therefore, bacteriophage YZ2 particles do not contain lipids and are chloroform-tolerant.

Therefore, phage YZ2 exhibited broad tolerance to variations in temperature, pH, and chloroform concentration, facilitating its preparation, storage, and potential clinical application.

### 3.2 Optimal number of infection replicates and one-step growth curve of phage YZ2

**Figure 3:**
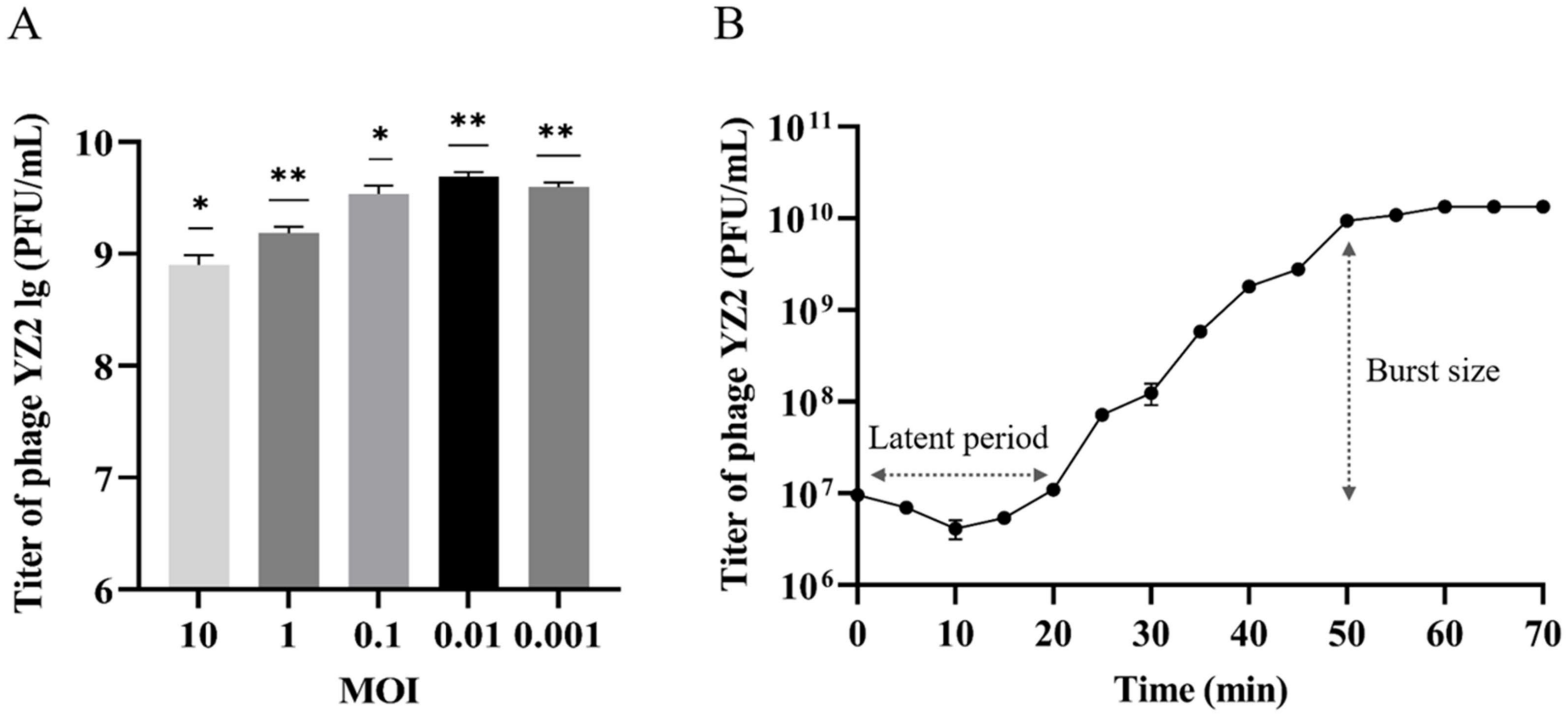
Biological characteristics of phage YZ2. **(A)** Determination of the optimal MOI for phage YZ2; **(B)** One-step growth curve of phage YZ2. Data are presented as mean ± SD (*p < 0.05, **p < 0.01, ***p < 0.001).

Phage concentrations at various MOIs were assessed using the double-layer agar plaque method. Phage YZ2 was combined with host *A. baumannii* at different MOIs. According to the results, YZ2 reached its highest titer of 4.9 × 10^9^ PFU/mL at an MOI of 0.01, which was considerably higher compared to other MOIs, showing that 0.01 is the optimal MOI for phage YZ2 (Figure 3A). A one-step growth curve assay was performed to determine the infection kinetics of phage YZ2. The specific time points for the one-step growth experiment (0, 5, 10, 15, 20, 25, 30, 35, 40, 45, 50, 55, 60, 65, and 70 min) were selected based on previous studies, with minor modifications (Pajunen et al., 2000). Single-cycle replication analysis demonstrated that YZ2 has a 20-min latent period followed by 30 min of active virion release, yielding an average progeny production of 134 plaque-forming units per infected host cell. After 50 min, the phage entered a slow-growth plateau phase (Figure 3B), showing kinetics similar to those of the phage vB_AbaP_D2 (Yuan et al., 2020).

### 3.3 Host range and lysis efficiency of phage YZ2

The host range of phage YZ2 was evaluated using 26 clinical CRAB strains from different sources, including *Escherichia coli* ATCC25922, *Klebsiella pneumoniae* ATCC700603, and *Pseudomonas aeruginosa* ATCC27853. TThe results of the spot assay obtained at 37 °C showed that phage YZ2 specifically infected all ST540 strains and 75% (6/8) of the ST208 strains (Supplementary Figure 1). Notably, YZ2 formed Clear lytic zones on the four ST540 strains (CRAB 008, CRAB 012, CRAB 014, and CRAB 016), while forming turbid lytic zones on the remaining strains. To further assess the lytic ability of YZ2 against CRAB, the plating efficiency was tested. EOP was categorized as high (EOP ≥ 0.5), moderate (0.5 > EOP ≥ 0.1), low (EOP ≤ 0.1), or inactive (EOP < 0.001) based on reproducible infectivity against target bacteria (Wintachai et al., 2022a). Subsequent titration revealed that phage YZ2 established highly productive infections (EOP = 0.88–1.09) in four out of seven ST540-type CRAB strains (Table 1), and halo rings were observed surrounding the formed plaques (Supplementary Figure 1), suggesting the presence of depolymerase activity. Phage YZ2 did not exhibit any lytic activity against *Escherichia coli* ATCC25922, *Klebsiella pneumoniae* ATCC700603, or *Pseudomonas aeruginosa* ATCC27853.

**Table 1:** Host range spectrum of the phage YZ2.

Strains	Source	ST type	Lysed or not^a^	EOP
CRAB 001	Sputum	ST 195	−	−
CRAB 002	Sputum	ST 208	+	<0.001
CRAB 003	Sputum	ST 540	+	<0.001
CRAB 004	Sputum	ST 195	−	−
CRAB 005	Sputum	ST 437	−	−
CRAB 006	Sputum	ST 540	+	<0.001
CRAB 007	Sputum	ST 208	+	<0.001
CRAB 008	Ascites	ST 540	++	0.95
CRAB 009	Sputum	ST 369	−	−
CRAB 010	Sputum	ST 369	−	−
CRAB 011	Sputum	ST 208	+	<0.001
CRAB 012	Blood	ST 540	++	1.09
CRAB 013	Sputum	ST 208	+	<0.001
CRAB 014	Sputum	ST 540	++	0.88
CRAB 015	Sputum	ST 208	−	−
CRAB 016	Sputum	ST 540	++	0.96
CRAB 017	Sputum	ST 208	−	−
CRAB 018	Wound exudate	ST 1482	−	−
CRAB 019	Sputum	ST 369	−	−
CRAB 020	Sputum	ST 195	−	−
CRAB 021	Throat swab	ST 208	+	<0.001
CRAB 022	Sputum	ST 369	−	−
CRAB 023	urine	ST 208	+	< 0.001
CRAB 024	Urine	ST 540	+	<0.001
CRAB 025	Urine	ST 844	−	−
CRAB 026	Urine	ST 1791	−	−
*P. aeruginosa* ATCC27853			−	−
*E. coli* ATCC25922			−	−
*K. pneumoniae* ATCC700603			−	−

a. ++, Clear lytic zone; +, Turbid lytic zone; −, No lytic zone formed.

### 3.4 Genome analysis and annotation of phage YZ2

The biological properties of bacteriophages and their treatment safety can be predicted using bioinformatics analyses. A range of bioinformatics tools have been employed to sequence and analyze the genome of phage YZ2 in detail. The complete linear double-stranded DNA genome spanned 40,181 base pairs with a GC content of 37.93%. To confirm the assembly sequence of the YZ2 genome, the purified YZ2 genome (which contains a single recognition-cutting site) was digested using the *Eco*RV restriction enzyme (Supplementary Figure 2). After enzymatic digestion, a single DNA band was produced, which initially suggested that the genome was a circular double-stranded DNA. However, we believe that its packaging form in the phage particles should be linear. Tailed phages usually cut the precursor tandem DNA into unit-length linear chromosomes before packaging (Casjens, 2011), and the YZ2 genome encodes a typical terminase. Therefore, the circular DNA we observed might have resulted from the linear genome of the phage being connected through sticky ends within the phage, or from circularization that occurred during DNA purification.

**Figure 4:**
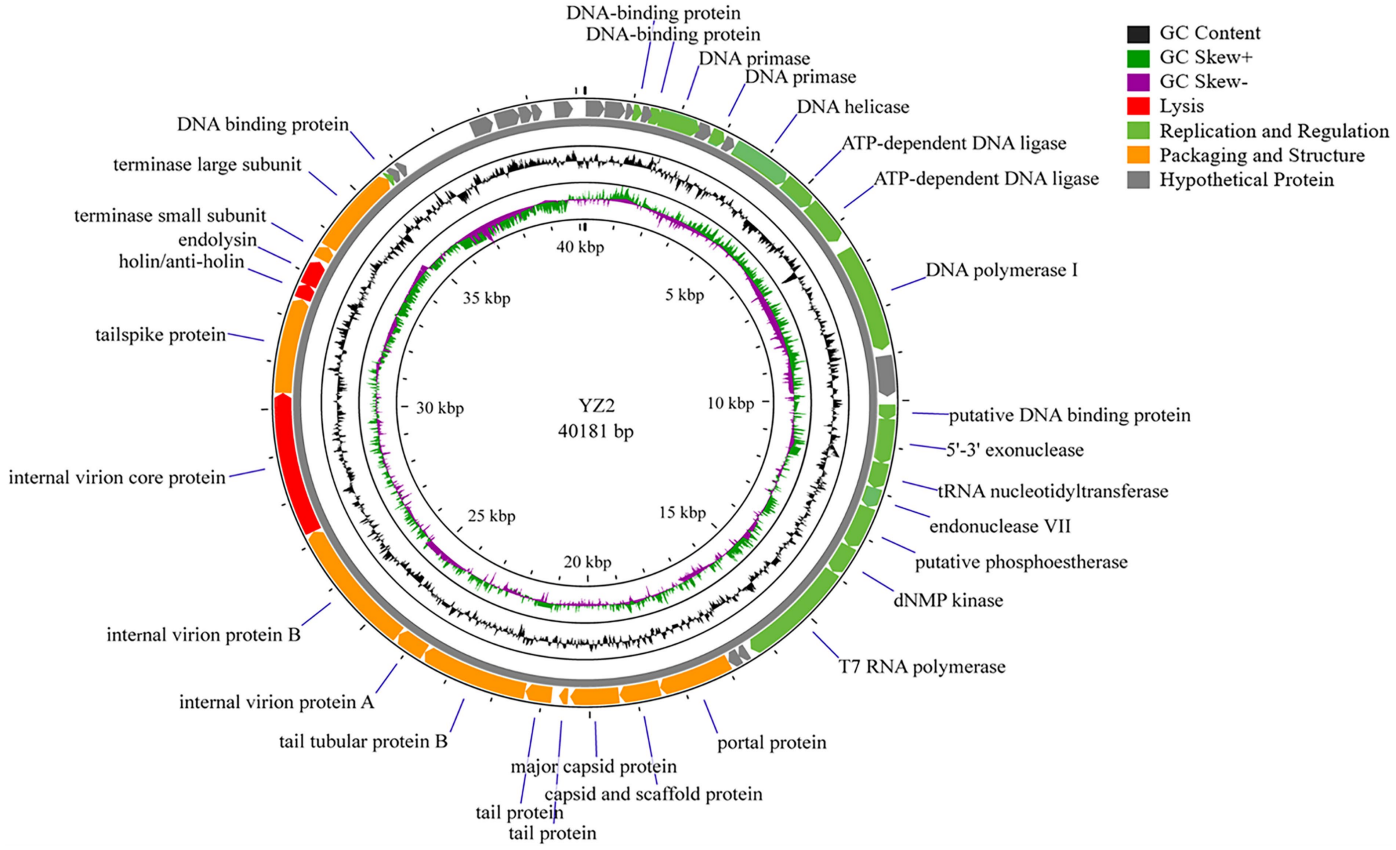
Schematic representation of the genomic organization of phage YZ2. Each predicted ORF is depicted as an arrow, with color coding to indicate functional categories: red, lysis-related genes; green, replication and regulatory genes; orange, packaging and structural genes; gray, hypothetical proteins. The black line represents GC content, with dark green indicating regions where (G − C)/(G + C) > 0 and purple indicating regions where (G − C)/(G + C) < 0.

Genome annotation using Prokka and BLASTP identified 46 predicted open reading frames (ORFs). Among these, 30 ORFs were functionally characterized in the NCBI database and encoded phage-associated proteins, including (i) lytic enzymes, (ii) replication and regulatory proteins, and (iii) packaging and structural components (Figure 4, Table 2). The remaining 16 ORFs were annotated as hypothetical proteins or showed no significant homology, thus warranting further investigation. Notably, genomic analysis revealed the absence of antibiotic resistance genes, virulence factors, and lysogenic cycle-associated genes (e.g., repressors or integrases). Phage YZ2 is speculated to be a lytic phage based on genome information from current databases and gene annotation. Importantly, no known virulence determinants were identified in the YZ2 genome. The lack of genes encoding pathogenic factors makes this lytic phage a promising and safe candidate for antimicrobial therapy.

**Table 2:** Host predicted ORFs encoded by phage YZ2 genome (GenBank accession: OR660046).

ORF	Start	Stop	Length (AA)	Putative function and best-match BLASTp result	PI	MW (kDa)	Accession No.	Identity (%)	Query cover (%)	*E*-value
ORF1	14	415	133	Hypothetical protein [*Acinetobacter* phage vB_AbaP_APK14]	9.13	14.80	AYR04356.1	100	100	4.90E−90
ORF2	417	854	145	Hypothetical protein [*Acinetobacter* phage AbpL]	9.07	16.46	UVD42100.1	100	100	6.05E−103
ORF3	865	1,032	55	Hypothetical protein [*Acinetobacter* phage IME-200]	10.15	6.81	YP_009216508.1	100	100	1.14E−25
ORF4	1,019	1,210	63	DNA-binding protein [*Acinetobacter* virus vB_AbaP_AGC01]	9.69	7.14	QIW86330.1	98.4	100	1.06E−40
ORF5	1,207	1,425	72	Hypothetical protein [*Acinetobacter* phage vB_AbaP_APK48-3]	9.05	7.92	QGH71536.1	100	100	1.83E−45
ORF6	1,367	1,639	90	DNA-binding protein [*Acinetobacter* phage AbpL]	9.47	10.19	UVD42104.1	81.1	100	1.00E−46
ORF7	1,629	2,429	266	DNA primase [*Acinetobacter* phage IME-200]	8.56	30.59	YP_009216512.1	98.1	100	0
ORF8	2,432	2,746	104	Hypothetical protein [*Acinetobacter* phage vB_Ab4_Hep4]	6.57	11.71	UVD33013.1	94.2	100	2.00E−66
ORF9	2,746	3,063	105	DNA primase [*Acinetobacter* phage Acba_6]	4.36	11.75	WCF71606.1	98.1	100	1.00E−67
ORF10	3,063	3,305	80	Hypothetical protein [*Acinetobacter* phage IME-200]	9.15	9.08	YP_009216514.1	100	100	3.00E−51
ORF11	3,318	4,616	432	DNA helicase [*Acinetobacter* phage IME-200]	5.44	48.37	YP_009216515.1	100	100	0
ORF12	4,613	5,356	247	ATP-dependent DNA ligase [*Acinetobacter* phage AbpL]	8.93	28.07	UVD42109.1	95.9	99	1.00E−176
ORF13	5,353	6,330	325	ATP-dependent DNA ligase [*Acinetobacter* phage vB_AbaP_ZHSHW]	5.9	36.53	UPT53538.1	98.5	100	0
ORF14	6,579	8,891	770	DNA polymerase I [*Acinetobacter* phage vB_AbaP_APK89]	5.48	87.78	QGK90371.1	99.2	100	0
ORF15	9,024	9,914	296	hypothetical protein [*Acinetobacter* phage vB_AbaP_APK81]	5.01	32.52	QNO11397.1	100	100	0
ORF16	10,087	10,419	110	Putative DNA binding protein [*Acinetobacter* phage vB_AbaP_B09_Aci08]	9.33	13.24	YP_009814041.1	98.2	100	7.00E−75
ORF17	10,400	11,368	322	5′–3′ exonuclease [*Acinetobacter* phage APK127v]	5.72	36.40	URQ05171.1	98.4	100	0
ORF18	11,358	11,927	189	tRNA nucleotidyltransferase [*Acinetobacter* phage vB_AbaP_ABWU2101]	4.96	21.54	UFJ83448.1	94.2	100	2.00E−129
ORF19	11,924	12,364	146	Endonuclease VII [*Acinetobacter* phage vB_AbaP_B1]	9.77	16.61	YP_009610315.1	100	100	6.00E−105
ORF20	12,368	13,303	311	Putative phosphoestherase [*Acinetobacter* phage vB_AbaP_B09_Aci08]	7.76	35.54	YP_009814045.1	99.7	100	0
ORF21	13,303	13,953	216	dNMP kinase [*Acinetobacter* phage vB_AbaP_AS12]	4.97	24.83	YP_009599215.1	99.5	100	9.86E−155
ORF22	13,962	16,379	805	RNA polymerase [*Acinetobacter* phage vB_AbaP_APK2-2]	6.07	90.93	AZU99279.1	99.9	100	0
ORF23	16,483	16,680	65	Hypothetical protein [*Acinetobacter* phage vB_AbaP_B3]	6.18	7.76	YP_009610367.1	98.46	100	4.00E−39
ORF24	16,677	16,928	83	Hypothetical protein [*Acinetobacter* phage vB_AbaP_B1]	6.21	9.06	YP_009610320.1	98.8	100	1.00E−52
ORF25	16,937	18,493	518	Head-tail adaptor [*Acinetobacter* phage vB_AbaP_AS11]	4.97	58.87	YP_009599271.1	100	100	0
ORF26	18,502	19,362	286	Capsid and scaffold protein [*Acinetobacter* phage vB_AbaP_PMK34]	5.25	30.54	QGF20166.1	100	100	0.00E+00
ORF27	19,378	20,409	343	Major capsid protein [*Acinetobacter* phage AbpL]	5.42	38.48	UVD42127.1	100	100	0.00E+00
ORF28	20,463	20,648	61	Tail protein [*Acinetobacter* phage IME-200]	4.65	6.44	YP_009216534.1	100	100	9.00E−33
ORF29	20,804	21,364	186	Tail protein [*Acinetobacter* phage AbKT21phiIII]	9.49	21.68	YP_009818752.1	100	100	1.00E−132
ORF30	21,373	23,664	763	Tail tubular protein B [*Acinetobacter* phage vB_AbaP_APK81]	4.9	84.20	QNO11414.1	99.1	100	0
ORF31	23,676	24,338	220	Internal virion protein A [*Acinetobacter* phage vB_AbaP_APK81]	8.8	22.99	QNO11415.1	100	100	3.00E−155
ORF32	24,351	27,236	961	Internal virion protein B [*Acinetobacter* phage vB_AbaP_APK2-2]	7.25	105.83	AZU99290.1	99.7	100	0
ORF33	27,246	30,344	1,032	Internal virion core protein [*Acinetobacter* phage BM12]	5.77	114.06	UYE92399.1	99.6	100	0
ORF34	30,351	32,432	693	Tailspike protein [*Acinetobacter* phage vB_AbaP_APK2]	5.14	75.25	AZU99242.1	99.9	100	0
ORF35	32,445	32,780	111	Holin/anti-holin [*Acinetobacter* phage IME-200]	5.12	11.94	YP_009216490.1	100	100	3.00E−73
ORF36	32,767	33,324	185	Endolysin [*Acinetobacter* phage IME-200]	9.54	21.19	YP_009216491.1	100	100	8.00E−131
ORF37	33,385	33,693	102	Terminase small subunit [*Acinetobacter* phage vB_AbaP_PD-AB9]	4.53	11.08	YP_009189827.1	100	100	2.00E−66
ORF38	33,703	35,640	645	Terminase large subunit [*Acinetobacter* phage AbpL]	6.54	72.80	UVD42138.1	99.7	100	0
ORF39	35,637	35,771	44	DNA binding protein [*Acinetobacter* phage IME-200]	8.1	5.13	YP_009216494.1	100	100	2.00E−21
ORF40	35,731	35,934	67	Hypothetical protein [*Acinetobacter* phage phiAB1]	9.85	7.00	YP_009189385.1	100	100	6.00E−36
ORF41	35,945	36,124	59	Hypothetical protein [*Acinetobacter* phage SH-Ab 15,519]	4.57	6.85	YP_009598261.1	100	100	5.98E−34
ORF42	37,726	38,196	156	Hypothetical protein [*Acinetobacter* phage IME-200]	4.36	17.36	YP_009216497.1	100	100	1.98E−111
ORF43	38,267	38,785	172	Hypothetical protein [*Acinetobacter* phage vB_AbaP_PD-AB9]	6.52	18.92	YP_009189871.1	99.4	97	3.00E−117
ORF44	38,782	39,054	90	Hypothetical protein [*Acinetobacter* phage AbKT21phiIII]	8.05	9.87	YP_009818724.1	62.6	100	7.00E−31
ORF45	39,056	39,265	69	Hypothetical protein [*Acinetobacter* phage vB_AbaP_APK81]	9.1	7.79	QNO11373.1	97.9	70	2.38E−25
ORF46	39,524	39,922	132	Hypothetical protein [*Acinetobacter* phage vB_AbaP_ABWU2101]	6.96	14.81	UFJ83470.1	100	100	6.53E−94

**Figure 5:**
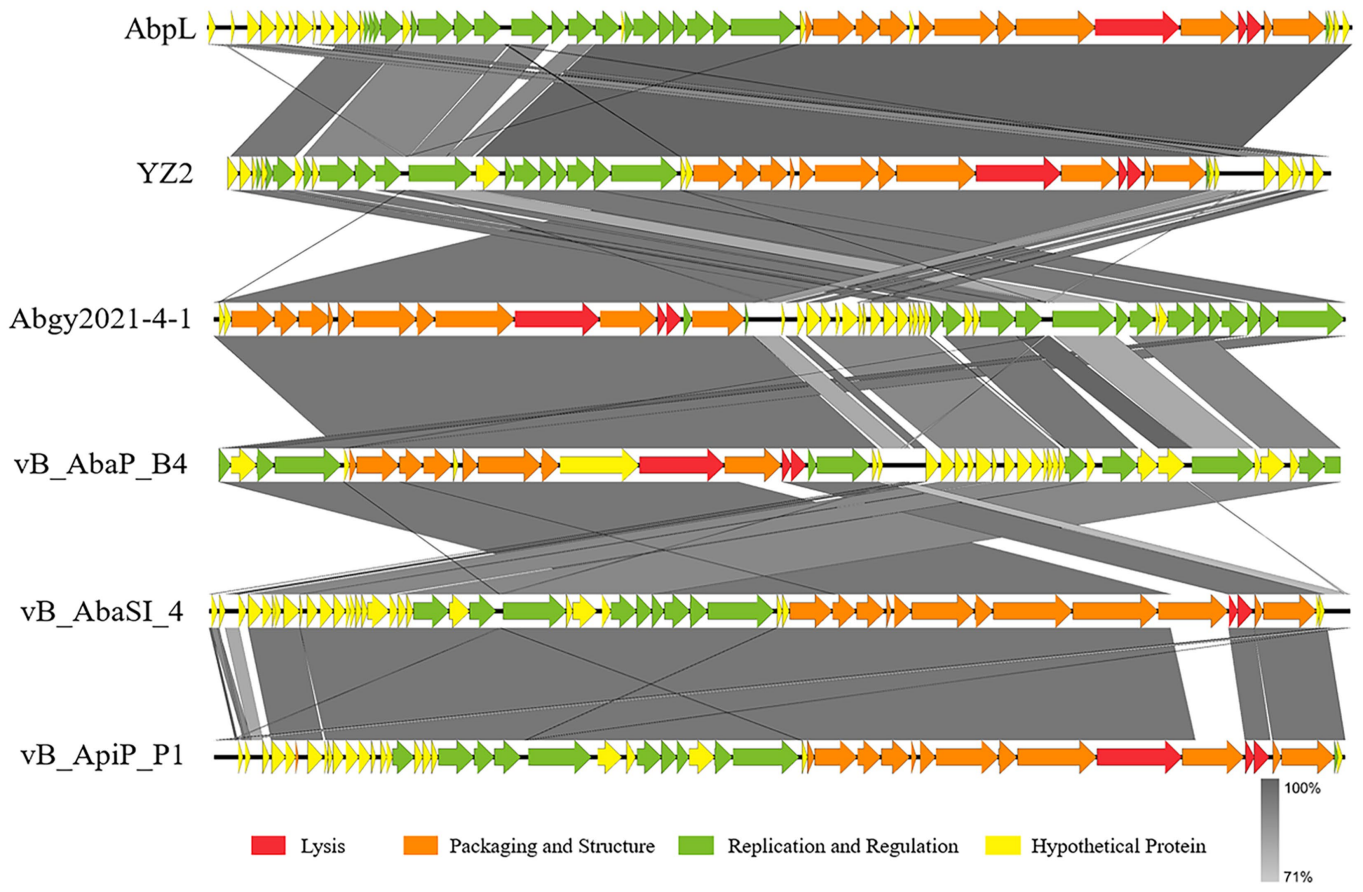
Alignment of multiple bacteriophage genome sequences. The whole-genome comparison of bacteriophage YZ2 with *Acinetobacter* phages AbpL, Abgy2021-4-1, VB_AbaP_B4, VB_AbaSI_4, and VB_ApiP_P1 was performed using Easyfig. Color-coded arrows represent complete CDSs across the genome sequences, gray shadows represent the level of sequence similarity between genomes.

BLAST analysis revealed that the YZ2 genome showed significant homology with several previously reported phages belonging to the same genus, including *Acinetobacter* phages AbpL, Abgy2021-4-1, VB_AbaP_B4, VB_AbaSI_4, and VB_ApiP_P1. The high query coverage (92–97%) and sequence identity (95.03–96.73%) fully confirm that YZ2 is a new member of this phage group. Comparative genomic analysis by EasyFig demonstrated a conserved modular organization among the six phages, particularly in the functional modules related to DNA replication/transcription, lysis, and structural assembly. While most proteins encoded by YZ2 were highly conserved in homologous phages (Figure 5), DNA polymerase I (ORF14) exhibited notable divergence: 87.69% identity (59% coverage) with AbpL and 81.15% identity (99% coverage) with Abgy2021-4-1. This variation likely arose from horizontal gene transfer (HGT) or functional domain recombination, reflecting an evolutionary strategy for host adaptation(Petrov et al., 2010). Critically, insertion/deletion polymorphisms in the functional genes and distinct genomic architectures confirmed that YZ2 represents a novel phage lineage. The bacteriophage YZ2 genome encodes key functional modules for replication, morphogenesis, and host lysis. DNA replication and transcription are facilitated by a suite of proteins, including DNA polymerase I (ORF14), helicase (ORF11), and T7 RNA polymerase (ORF22), supported by ancillary factors for DNA repair (e.g., exonuclease ORF17 and endonuclease ORF19) and nucleotide metabolism. The virion is composed of a capsid built from major capsid protein (MCP, ORF27) and a tail structure featuring a host-recognition tail-spike protein (ORF34). Host lysis is achieved through the coordinated action of holin (ORF35) and endolysin (ORF36).

**Figure 6:**
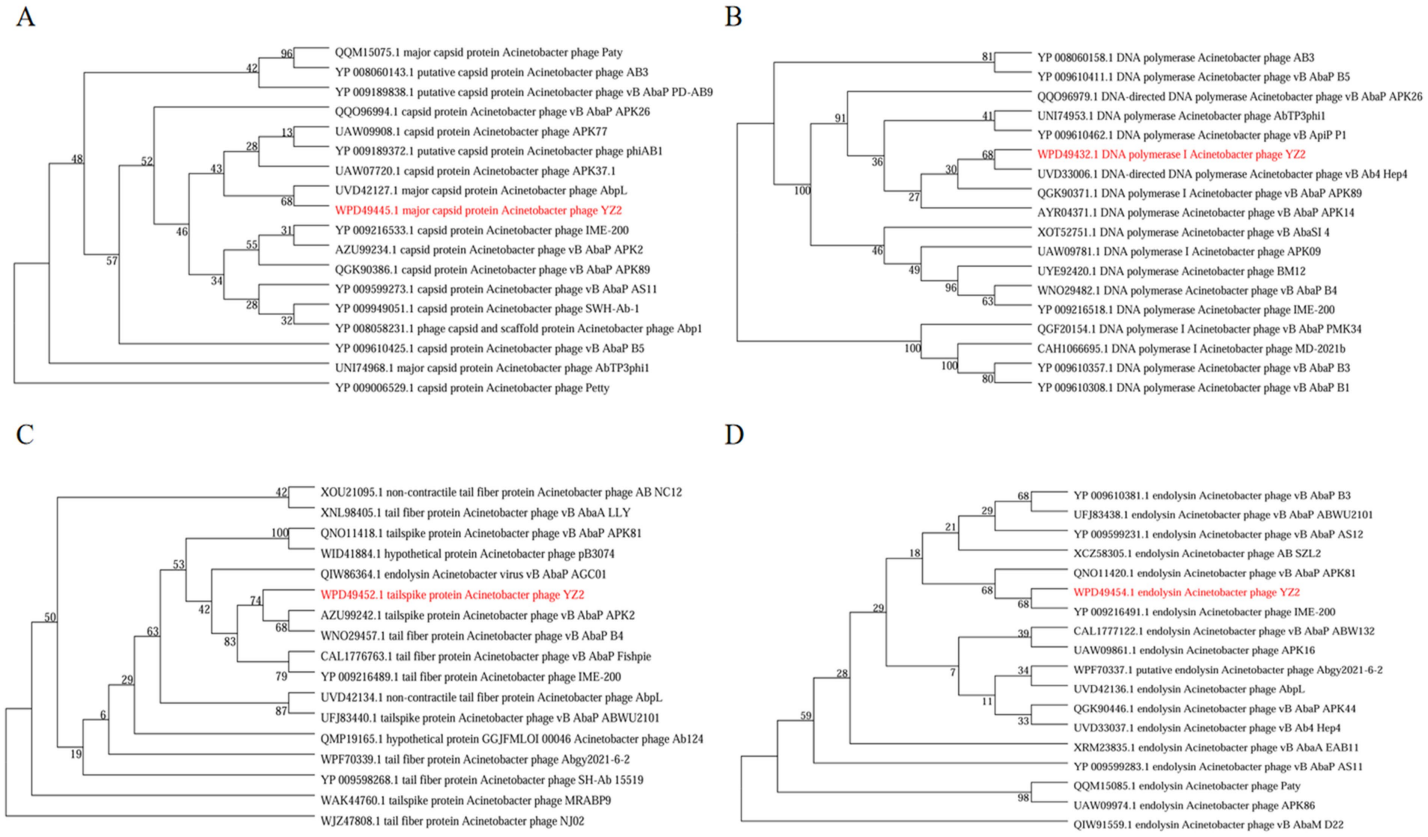
The evolutionary tree of particular amino acid sequences in phages. **(A)** Phylogenetic tree created from major capsid protein sequences, **(B)** tree assembled with DNA polymerase I sequences, **(C)** phylogeny based on tail spike protein sequences, and **(D)** tree generated from endolysin sequences. *A. baumannii* phage YZ2 is highlighted in red. Bootstrap analysis with 1,000 replicates was performed to assess the reliability of the phylogenetic tree.

To elucidate the evolutionary relationships between bacteriophages, we constructed phylogenetic trees based on conserved protein sequences. Reference sequences were retrieved from standard databases using Blastp, followed by comparative analysis using MEGA v7.0 software (Kumar et al., 2016). Phylogenetic analysis identified four key functional proteins from phage YZ2: MCP, DNA polymerase I, tail spike protein, and endolysin. Maximum-likelihood phylogenetic reconstruction revealed that: The MCP of phage YZ2 exhibited 100% sequence identity with *Acinetobacter* phage AbpL (Figure 6A); The DNA polymerase I showed 95.38% nucleotide sequence identity with *Acinetobacter* phage vB_Ab4_Hep4 (Figure 6B); The tailspike protein demonstrated 99.86% sequence identity with *Acinetobacter* phage vB_AbaP_APK2 (Figure 6C); The endolysin displayed complete (100%) sequence identity with *Acinetobacter* phage IME200 (Figure 6D). Molecular phylogenetic analysis demonstrated that phage YZ2 exhibits the closest evolutionary relationship to the four aforementioned *Acinetobacter* phages, particularly in terms of their structural and lytic proteins. All analyzed proteins showed high sequence conservation (>95% identity), suggesting that these phages shared a relatively recent common ancestor.

### 3.5 Subsection evaluation of *in vitro* and *in vivo* efficacy of phage YZ2 against *A. baumannii* AB0814

**Figure 7:**
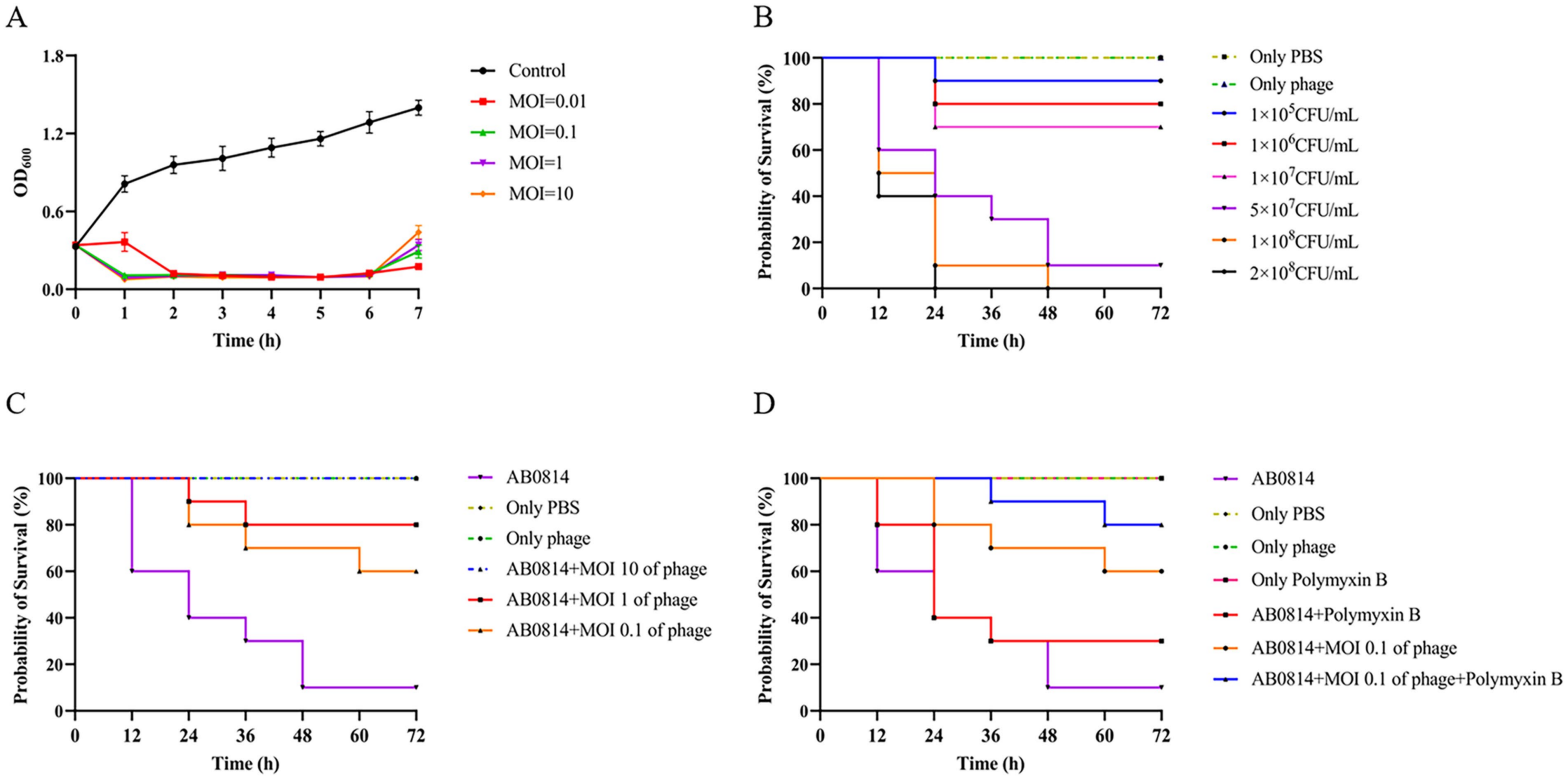
Evaluation of *in vitro* and *in vivo* therapeutic effects. **(A)** The ability of phage YZ2 to lyse *A. baumannii* AB0814 at various MOIs; **(B)** Survival curves for *G. mellonella* larvae following injection with different concentrations of *A. baumannii* AB0814; **(C)** Survival rates for *G. mellonella* larvae post-treatment with phage YZ2 after *A. baumannii* AB0814 infection; **(D)** Survival rates of *G. mellonella* larvae treated with combined phage YZ2 and antibiotic therapy against *A. baumannii* AB0814 infection.

Using uninfected *A. baumannii* AB0814 as the control, the lytic activity of phage YZ2 was measured for 7 h at different MOIs (10, 1, 0.1, and 0.01). The results showed that at different MOIs, the optical density (OD) of bacterial cultures in all phage treatment groups decreased significantly within 1 h after infection, whereas the OD of the non-infected control group increased continuously. This low OD state persisted for up to 6 h (Figure 7A). Subsequent OD increases in the phage-treated groups between 6–7 h indicated the emergence of phage-resistant bacteria.

**Figure 8:**
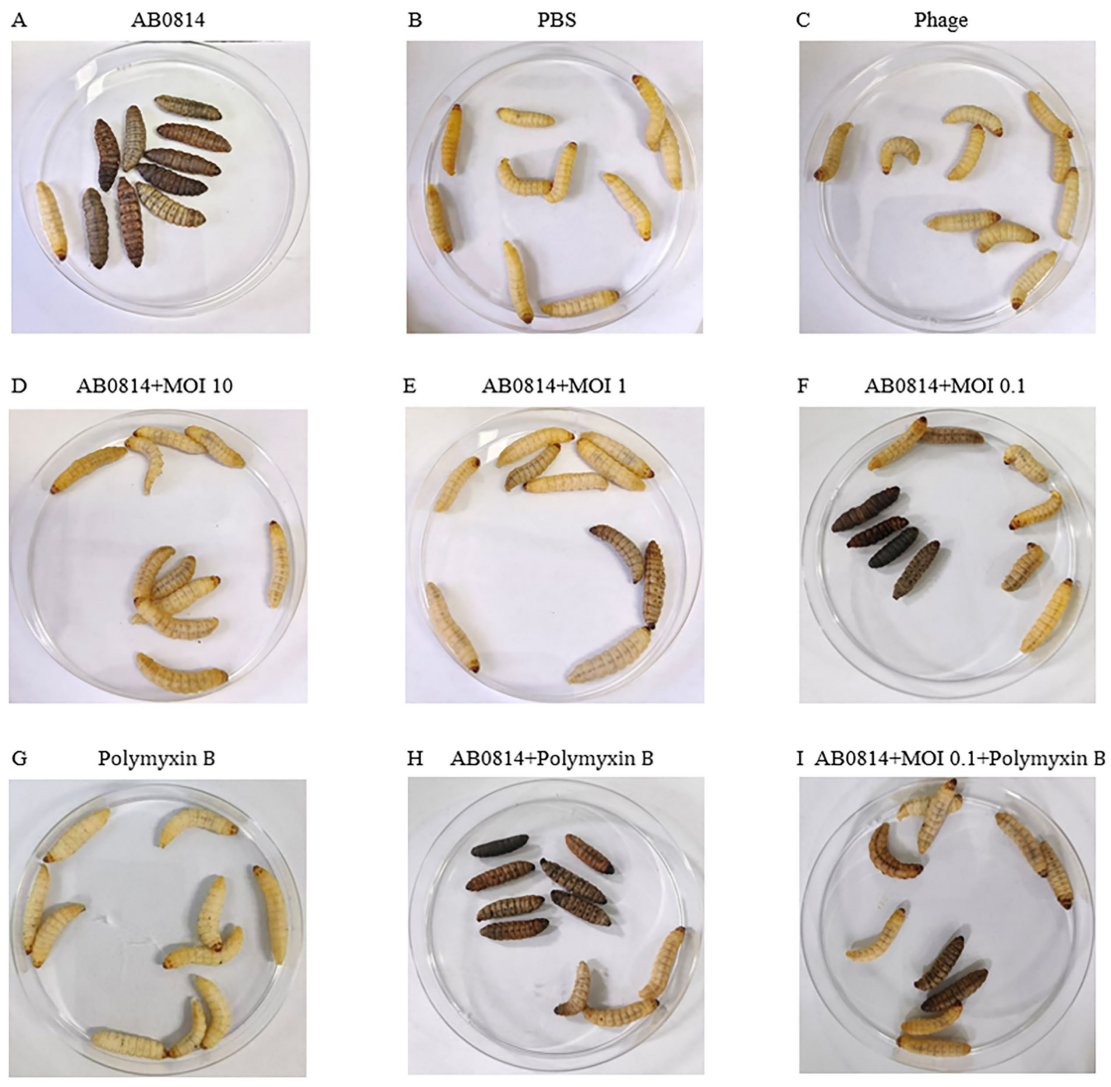
Morphological characteristics of *G. mellonella* larvae. Viable larvae exhibit a light yellow coloration without spots, whereas dead larvae appear black. **(A)** Only AB0814; **(B)** Only PBS; **(C)** Only phage; **(D-F)** Phage YZ2 treatment post AB0814 infection; **(G)** Only Polymyxin B; **(H)** Polymyxin B treatment post AB0814 infection; **(I)** Combination therapy with phage YZ2 and Polymyxin B.

The effectiveness of YZ2 treatment was assessed *in vivo* using a *G. mellonella* larval model. The survival curves following injection with different AB0814 concentrations identified 5 × 10^7^ CFU/mL as the optimal challenge dose, which caused progressive larval mortality within 72 h (Figure 7B). In control experiments: 90% mortality occurred in the AB0814-infected positive control larvae after 72 h (Figure 8A). Both the PBS-injected and phage-only (MOI = 10) groups maintained 100% survival (Figures 8B and 8C). These findings confirmed that neither PBS nor phage YZ2 alone induced larval mortality, demonstrating the excellent biosafety of this phage preparation. The excellent biosafety of this phage preparation was further corroborated by its low endotoxin concentration (57 EU/10^9^ PFU).

Evaluation of the therapeutic efficacy of phage YZ2 as a potential antimicrobial agent demonstrated dose-dependent protection of *G. mellonella* larvae, with survival rates of 100, 80, and 60% at MOIs of 10, 1, and 0.1, respectively (Figures 7C and 8D-F). A positive relationship was seen between the phage titer (MOI) and the survival rate of larvae, with all phage-treated groups showing significant protection compared with the untreated controls. Notably, the highest MOI (10) group exhibited optimal therapeutic outcomes, achieving complete (100%) survival protection, indicating the potent *in vivo* antibacterial activity of phage YZ2 at elevated concentrations.

The combination of antibiotics and phages has been studied both *in vitro* and in animal models(Segall et al., 2019). Therefore, in this study, an antibiotic-phage combination was used to treat AB0814 infection in *G. mellonella* larvae. The injection of polymyxin B alone did not cause larval mortality (Figures 7D and 8G). When infected larvae were treated with the phage at an MOI of 0.1 or polymyxin B alone, the survival rates at 72 h were 60 and 30%, respectively (Figures 7D, 8F, and H). However, the combination of YZ2 and polymyxin B ensured an 80% larval survival rate within 72 h (Figures 7D and 8I). These results confirm that phage YZ2 not only effectively inhibits AB0814 growth, but also enhances therapeutic efficacy when combined with antibiotics, providing important evidence for the development of clinical combination therapy strategies.

## 4 Discussion

Antibiotic resistance is a major public health concern worldwide(Luo et al., 2024b). The rise in bacterial resistance to multiple drugs has resulted in a critical lack of effective antibiotics for clinical use. The rising resistance to carbapenem antibiotics, which are considered the ultimate treatment for multidrug resistant Gram-negative infections, represents a particularly pressing issue (Papp-Wallace et al., 2011). According to CDC data, the prevalence of MDR in *A. baumannii* (AB) is four-fold higher than that in other Gram-negative bacteria, such as *P. aeruginosa* and *K. pneumoniae* (Giammanco et al., 2017), drug resistance in AB is correlated with increased mortality and poses significant challenges to effective clinical treatment (Ibrahim et al., 2021). In recent years, escalating bacterial resistance, emergence of carbapenemases, and lack of novel antibiotics have raised grave concerns, underscoring the urgent need for well-tolerated and efficient therapeutic alternatives (Castanheira et al., 2017). This has prompted researchers to revisit bacteriophages as complementary or alternative agents to combat resistant bacterial infections (Anyaegbunam et al., 2022).

In this study, we discovered a new phage, named YZ2, which created large clear zones with haloes on the host bacterial lawn, suggesting the existence of a bacteriophage-encoded depolymerase that can break down capsular polysaccharides (Knecht et al., 2020). Phage stability and activity are prerequisites for therapeutic applications (Pires et al., 2020). Compared to AB phage Ab_WF01 (Wang et al., 2024d), YZ2 demonstrated superior stability across temperatures (4–60 °C). Its pH tolerance (pH 5–11) exceeds that of AB phage PMK34 (Abdelkader et al., 2022), with notable resilience under alkaline conditions. YZ2 remained stable after chloroform treatment. Burst size and latency period are critical parameters for phage therapy (Gao et al., 2022), and YZ2 has a brief incubation period and a significant outbreak size (134 PFU/infected cell), making it a potential candidate for treating CRAB infections. The growth plateau is the standard time for harvesting high-titer phage lysate, which can provide high-yield and high-titer preparations (Kropinski, 2018). Nonetheless, the use of such lysates carries inherent risks. The primary concerns are the presence of substantial amounts of bacterial residual debris and endotoxins, which may trigger the host’s immune response (Raetz and Whitfield, 2002), as well as the potential inclusion of drug-resistant mutant strains that survive the lysis cycle. Therefore, in the future, we will adopt more efficient purification methods to remove endotoxins, such as combining ultrafiltration with the EndoTrap HD adsorption column (Hietala et al., 2019).

The host range is another vital therapeutic criterion. EOP provides a stricter evaluation of the host range because, with fewer phages, plaques can only develop through successful infection, which involves cell lysis and the release of viable progeny virions. The lysis zones observed under spot assay conditions may arise from outer membrane destabilization caused by the adsorption of a large number of phages, aligning with the classic definition of ‘lysis from without’ (Abedon, 2014). Alternatively, they could result from abortive infections in which the phage replication process is suppressed (Labrie et al., 2010). Phage infection specificity is highly complex, contingent on intricate interactions at each stage of the lifecycle (de Jonge et al., 2019). The narrow host range of YZ2 is a clear manifestation of this specificity. Unlike broad-spectrum antibiotics, lytic phages exhibit strain-specific activity, minimizing the disruption of commensal microbiota (Zhang et al., 2013). The YZ2 strain exhibited potent and consistent lytic activity against the ST540-type CRAB isolate, but only had partial and variable efficacy against ST208. This difference is consistent with the known biological characteristics of these clones. ST208 has enhanced genomic plasticity and a richer set of mobile genetic elements, and may be more likely to evade lysis by receptor modification or acquisition of phage resistance genes (Luo et al., 2025). In contrast, the newly emerged ST540 strain, although possessing strong multidrug resistance properties (Huang et al., 2024), is highly sensitive to YZ2. Therefore, the bacteriophage YZ2 is an excellent candidate for targeting infections caused by the ST540 strain.

The genome of phage YZ2 is a complete linear double-stranded DNA measuring 40,181 bp in length, with a GC content of 37.93%. Phage YZ2 was deemed safe for potential applications, because genetic testing revealed no antibiotic resistance genes, virulence factors, or lysogenic genes. The YZ2 phage was found to be highly similar to the five other phages (*Acinetobacter* phages AbpL, Abgy2021-4-1, VB_AbaP_B4, VB_AbaSI_4, and VB_ApiP_P1) through comparative genomic analysis, with most genes showing significant homology. High sequence homology of functionally similar genes has been identified across different phages, demonstrating that HGT constitutes an integral component of phage evolution (Chen et al., 2016; Yosef et al., 2017). Notably, phage YZ2 exhibited distinct genomic organization compared to related phages, a phenomenon consistent with gene rearrangements previously observed in other *A. baumannii* phages (Wang et al., 2024b). These results imply that YZ2 and related strains likely developed through HGT and genomic rearrangement, which is a well-known evolutionary trend in tailed phages (Jeon et al., 2019; Tian et al., 2024; Wang et al., 2024d).

Phylogenetic analysis using sequences of the MCP, DNA polymerase I, caudal spike protein, and endolysin revealed that YZ2 shares higher sequence similarity with phage AbpL in the MCP than with phage IME200. Similarly, genomic sequence alignment of DNA polymerase I demonstrated the closest relationship between YZ2 and the *Acinetobacter* phage Vb_Ab4_Hep4. Previous studies have identified depolymerases in either tail fibers or tail spike proteins. Notably, TaPaz encodes two distinct depolymerases (TaPaz_gp78 and TaPaz_gp79) within its tail spike proteins that effectively degrade bacterial capsular polysaccharide (Shchurova et al., 2021). Phage IME200 encodes depolymerase Dpo48, which can degrade capsular polysaccharides, thereby sensitizing bacteria to serum (Liu et al., 2019). We hypothesized that the tail spike protein encoded by ORF34 in phage YZ2 might possess similar capsular depolymerase activity, given its high sequence identity (99.13% by sequence identity matrix) with the tail fiber protein of the *Acinetobacter* phage IME200, which encodes the depolymerase Dpo48. Endolysins, encoded by phages, are peptidoglycan hydrolases used by most dsDNA phages to degrade the cell wall peptidoglycan layer and release progeny phages (Soontarach et al., 2024). Endolysin Abtn-4 from *Acinetobacter* phage D2 effectively reduces biofilm formation (Yuan et al., 2020), and phylogenetic analysis of endolysins indicated that YZ2’s endolysin is closely related to that of *Acinetobacter* phage IME200. Phage YZ2 exhibited rapid adsorption kinetics, high adsorption efficiency, short replication cycles within host cells, and large burst sizes. *In vitro* antibacterial activity assessments demonstrated that YZ2 could rapidly eliminate host bacteria at a low MOI, indicating potent bactericidal activity. However, as shown in Figure 7A, the optical density of the bacterial culture began to rise again approximately 6 h after phage treatment. This regrowth phenomenon is common in phage therapy. The most frequent explanation is the emergence of phage-resistant mutants, which may arise from mutations in phage receptors or the acquisition of innate defense systems (Labrie et al., 2010; Costa et al., 2024). Alternatively, the system may have reached a dynamic equilibrium or a ‘phage-bacteria arms race’ state (Koskella and Brockhurst, 2014), where a slowdown in new phage production allows the bacterial population to recover. These observations indicate that while monophage therapy is effective, it faces the challenge of resistance evolution. This finding strongly supports the future development of phage cocktail therapies or phage-antibiotic combinations to suppress resistance and achieve a more persistent antibacterial effect.

The therapeutic potential of phage-antibiotic synergy against MDR bacterial infections has garnered increasing attention (Segall et al., 2019). The *G. mellonella* larval infection model has become a widely adopted system for evaluating phage efficacy (Tsai et al., 2016), offering the significant advantages of lower costs and minimal ethical concerns compared to other animal models (Tao et al., 2021). This model serves as a valuable platform for assessing the therapeutic potential of phages and their capacity to eradicate pathogens in complex biological environments as demonstrated in larger animal models (Wang et al., 2024d). Several studies have investigated phage-antibiotic combinations in *G. mellonella* larval models. *Acinetobacter* phage vB_AbaP_AGC01 demonstrated synergistic effects when combined with ciprofloxacin and meropenem (Grygorcewicz et al., 2020), whereas no consistent synergy was observed between *Acinetobacter* phages WCHABP1/WCHABP12 and polymyxin B (Zhou et al., 2018). In our infection experiments, phage YZ2 exhibited no toxicity towards *G. mellonella* larvae and showed remarkable therapeutic efficacy in this infection model. Importantly, the combination of phage YZ2 with polymyxin B proved to be more effective in eradicating CRAB than using each treatment separately, significantly improving larval survival rates. These findings strongly suggest that YZ2 has substantial clinical potential as a therapeutic agent.

This study found that the phage YZ2 and polymyxin B have a significant synergistic bactericidal effect on pan-drug-resistant *A. baumannii*. The mechanism of the bacteriophage-antibiotic combination is not fully understood. It is worth noting that bacteriophages can disrupt the structure of biofilms and enhance the penetration ability of antibiotics into the deep layers of biofilms by inducing the synthesis of enzymes such as polysaccharide depolymerases (Hanlon, 2007). It has been shown that mutant strains of *A. baumannii* lacking the capsule exhibit significantly enhanced sensitivity to colistin (Wang et al., 2021). Phages can be combined with various antibiotics to produce a significant synergistic bactericidal effect, and they can also effectively prevent the formation of drug-resistant strains through the ‘adjuvant effect’ (Gu Liu et al., 2020). Classic studies have confirmed that sub-inhibitory concentrations of penicillin can effectively promote the replication of bacteriophages and bacteriolytic activity while inhibiting bacterial growth (Krueger et al., 1948). Studies have shown that sub-inhibitory concentrations of antibiotics, such as meropenem, can promote the replication of bacteriophages by altering the morphology of host bacteria, significantly increasing the number of phage plaques and enhancing their titer (Kamal et al., 2015). Colistin can enhance the adsorption of bacteriophages and the injection of phage DNA by altering the structure of the bacterial outer membrane (Jansen et al., 2018). The observed synergistic effect in this study may be the result of multiple mechanisms working together. First, the bacteriophage YZ2 may degrade the capsule through its depolymerase, removing the physical barrier imposed by polymyxin B, which represents the ‘phage-promoting antibiotic’ pathway. Second, polymyxin B may also disrupt the outer membrane of *A. baumannii* AB0814, exposing more or more accessible phage receptors, thereby passively enhancing the adsorption and replication efficiency of phage YZ2. This is the ‘antibiotic-promoting phage’ pathway. These two mechanisms may coexist and reinforce each other, but the dominant mechanism still requires further study.

Although we observed significant therapeutic effects of the phage YZ2 alone and its combination with polymyxin B in the *G. mellonella* model, we must acknowledge the limitations of this study. While PEG precipitation effectively reduced endotoxin levels in our phage preparation (57 EU/10^9^ PFU), the possibility of minor contributions from other residual bacterial components cannot be entirely ruled out. However, the low endotoxin load suggests that the synergistic effect with polymyxin B is primarily mediated by the bacteriophages themselves. Future studies utilizing specialized affinity chromatography designed for high-efficiency endotoxin removal (e.g., EndoTrap HD) will be valuable for obtaining ultra-pure phages and conclusively confirming the specific role of phage virions in this interaction. Furthermore, since the *G. mellonella* model lacks the adaptive immune system that mammals possess (Ménard et al., 2021), the findings of this study ultimately need to be verified in mammalian infection models. Future research will focus on using the CRAB infection model in mice to evaluate the pharmacokinetic characteristics of the phage YZ2 and its actual efficacy in a more complex immune environment (Zampaloni et al., 2024). This is a crucial step towards clinical translation.

## 5 Conclusion

The newly isolated and characterized phage YZ2 demonstrates promising potential as a substitute antimicrobial agent for controlling CRAB infections, both *in vitro* and *in vivo*. Its high efficacy in the *G. mellonella* therapeutic model underscores its potential for clinical translation. The YZ2-antibiotic combination may represent a crucial strategy against CRAB infections. Further investigation is required for its clinical application, with subsequent evaluation of YZ2’s therapeutic efficacy in mammalian models.

## Data Availability

The datasets presented in this study can be found the NCBI (https://www.ncbi.nlm.nih.gov), GenBank accession OR660046 and BioProject accession PRJNA1276704.

## Footnotes

2
https://cge.food.dtu.dk/services/VirulenceFinder

3
http://stothard.afns.ualberta.ca/cgview_server/
