# Supplementary material for: Identification of multi-drug resistant Acinetobacter baumannii phage YZ2 and evaluation of its therapeutic efficacy in vivo and in vitro
Source: Front Microbiol. 2025 Nov 24;16:1657539. doi: 10.3389/fmicb.2025.1657539 (PMC12682814; doi:10.3389/fmicb.2025.1657539)
Supplement: Supplementary file 1 [file Supplementary_file_1.docx]

Supplementary Material

# Supplementary Table 1 The antibacterial spectrum of *A. baumannii* AB0814.

| Antibiotic | Mic (µg/mL) | Sensitivity |
| --- | --- | --- |
| Ampicillin-sulbactam (SAM) | >32/16 | R |
| Piperacillin-tazobactam (TZP) | >128/4 | R |
| Cefoperazone-sulbactam (SCF) | >32/8 | R |
| Colistin (CT) | ≤1 | S |
| Imipenem (IMP) | >8 | R |
| Meropenem (MEM) | >8 | R |
| Ceftazidime (CAZ) | >32 | R |
| Cefepime (FEP) | >32 | R |
| Amikacin (AMK) | >32 | R |
| Gentamicin (GEN) | >8 | R |
| Tobramycin (TOB) | >8 | R |
| Trimethoprim- sulfamethoxazole (SXT) | ≥4/76 | R |
| Nitrofurantoin (F) | >64 | R |
| Ciprofloxacin (CIP) | >4 | R |
| Levofloxacin (LEV) | >8 | R |
| Tetracycline (TE) | >8 | R |
| Minocycline (MH) | 4 | S |

^a^ R, resistant; S, sensitivity.

# Supplementary Figure 1


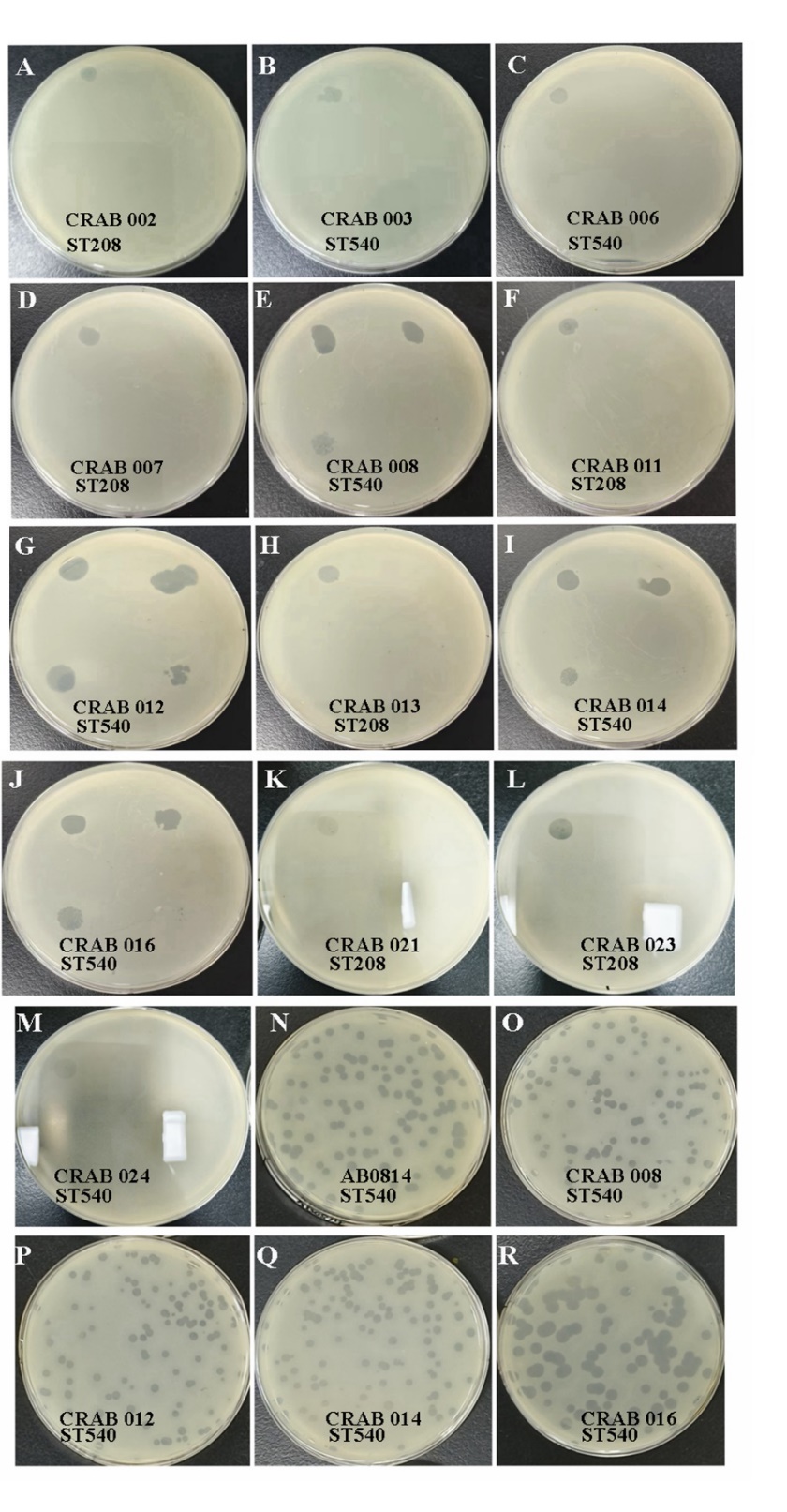


**Supplementary Figure 1.** **Host range spectrum of the phage YZ.** The assay was performed at 37 °C on a panel of CRAB clinical isolates. Lysis zones indicate susceptibility to the phage Figure 1 (A-M). The high efficiency of infection of the ST540 type CRAB isolate by the YZ2 phage is shown in Figure 1 as O to R. On the plates with the host bacteria CRAB 008, CRAB 012, CRAB 014, and CRAB 016, the phage YZ2 forms clear circular plaques with surrounding halos.

# Supplementary Figure 2


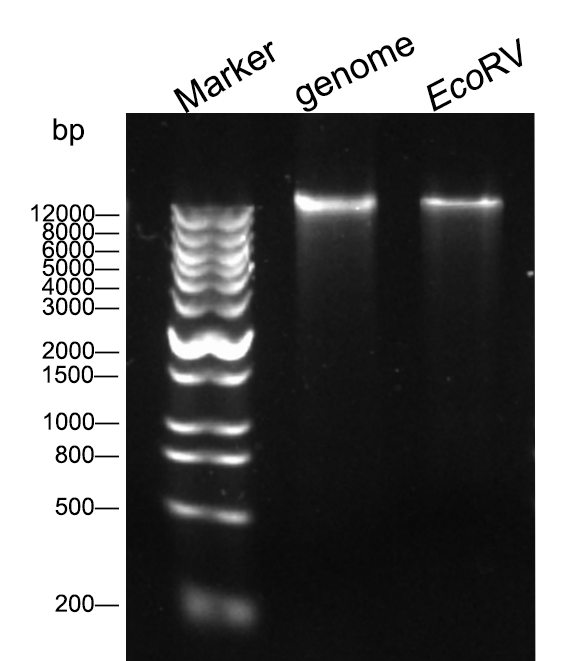


**Supplementary Figure 2. Restriction digestion profiles of the YZ2 genome.** The genome of YZ2 was digested with *Eco*RV. Marker: 12K DNA ladder marker.
